# Acute-Onset Cognitive Impairment Following COVID-19 Infection

**DOI:** 10.1192/bjo.2025.10111

**Published:** 2025-06-20

**Authors:** Jennifer Ford, Vinu Cherian

**Affiliations:** Essex Partnership University NHS Trust, Essex, United Kingdom

## Abstract

**Aims:** A recently published systematic review studying the link between COVID-19 and new-onset dementia (NOD) concluded that COVID-19 infection is likely to be a risk factor for developing NOD in older adults at 12 months, but no increased risk was noted at 3 or 6 month post-infection. We present a case of an elderly gentleman ‘X’ with no prior history of any cognitive deficit, who developed behavioural problems and cognitive decline within 6 weeks of a COVID-19 infection.

**Methods:** X is a 70-year-old gentleman with no previous history of psychiatric illness prior to March 2020, when he contracted COVID-19. Within 6 weeks of a positive swab, there was an acute change in his behaviour and cognition, wherein he was noted to be more talkative, disinhibited, presenting with grandiose ideas, sleeping very little at night and displaying intermittent episodes of confusion. X had never complained of problems with his memory and the family had never previously raised concerns about the same. However, cognitive testing carried out at 3 months post-infection revealed a significant decline in cognitive ability (Montreal Cognitive Assessment 17/30).

X was referred to the neurology team, which carried out a battery of tests including MRI scans, DAT scan, cerebrospinal fluid analysis and antibody screens. These largely came back negative and no organic cause could be determined for X’s presentation. However, the screen for Neurofilament Light Polypeptide (NFL) was positive, which was reported to possibly indicate damage following COVID-19 infection. NFL is known to have links with Alzheimer’s disease pathology. Cognitive tests repeated in 2024 have returned a MOCA score of 16/30, indicating that X’s cognitive impairment continues to persist.

**Results:** A retrospective cohort study by Taquet et al. which looked at the association between COVID-19 infection and psychiatric disorder found that there was a two to three times increased risk of dementia after COVID-19 infection. A subsequent larger study by the same authors published in Lancet Psychiatry which looked at 6-month neurological and psychiatric outcomes following COVID-19 infection confirmed the link between COVID-19 and risk of NOD. The presentation of our patient X is consistent with this finding.

**Conclusion:** Our case study highlights the increased risk of cognitive decline and NOD following COVID-19 infection in older adults.

